# An Apology to Dr. L. C. Bryan, of Basel, Switzerland

**Published:** 1904-02

**Authors:** F. Milton Smith

**Affiliations:** New York City


### Domestic Correspondence.

### AN APOLOGY TO DR. L. C. BRYAN, OF BASEL, SWITZERLAND.

To the Editor:

Sir,—Will you kindly permit me, through the Journal, to make my humble apology to Dr. L. C. Bryan for what appears to me, as I read it in cold type, a little discourteous.

Dr. Bryan in his reply (see January International Dental Journal, page 69) to the discussion at the meeting of the Institute of Stomatology, held May 5, 1903, upon his paper, which was read and discussed that evening, says, "Dr. F. Milton Smith says 'there is nothing new in either the paper of Dr. Bryan or that by Dr. Smith, of Philadelphia.'"

By reference to the discussion I read (International Dental Journal, November, 1903, page 857), "Dr. F. Milton Smith stated that it seemed to him that the whole thing was a matter of thoroughness. He could not see that there were any very new things suggested either by the paper of the evening or by Dr. Smith, of Philadelphia. It seemed to him that if Dr. Smith had announced that we were at times a little careless and that we ought to be more thorough, he would have hit the nail on the head. To this charge he [Dr. F. Milton Smith] would plead guilty."

My apology to Dr. Bryan is not for these remarks. I think any fair-minded man will see in them at least a little more courtesy than Dr. Bryan's *quotation* would seem to indicate.

Dr. Bryan also in his reply states that "it would be something new if a Dr. Smith of New York would acknowledge that a Dr. Smith of Philadelphia could tell him anything."

As to this, may I at this time say publicly that which I have spoken many, many times in private,—that through the courtesy of Dr. D. D. Smith, of Philadelphia, I was permitted to inspect, in his office, the mouths of probably ten or twelve people who had been under his treatment for some years. These patients represented almost every class of dental service. As an exhibition of magnificent *thoroughness* in cleansing and polishing, in gold fillings and bridge-work, and in amalgam (I think), in fact, in almost every class of work, I have never seen its equal; and I further say that I very much doubt if five men in the whole world can show such a beautiful result of thoroughness in all-around dental service, including oral prophylaxis.

This, of course, is only my opinion. In the sense that I had never seen such a beautiful dental exhibit before, I am compelled to say that Dr. Smith did show me something new.

To admit, however, that Dr. D. D. Smith has discovered an entirely new process, a sort of "Aladdin's lamp," so to speak, that by its magical power changes poor broken-down teeth into perfect glistening pearls, I am not willing to admit. I will admit that Dr. Smith has certainly provoked me to be more thorough and careful, and for this I am profoundly grateful to him.

But the remark for which I desire to apologize to Dr. Bryan is found on page 858 (INTERNATIONAL DENTAL JOURNAL), sixth and seventh lines: "He [Dr. F. Milton Smith] believed there were thorough men outside of Philadelphia and this side of Switzerland." This was certainly superfluous and, as it appears to me, not quite in tune with that high standard which every true gentleman should seek to attain unto.

I make my humble apology to Dr. Bryan, and promise him in the future I will try to be a more *thorough* gentleman.

F. MILTON SMITH.

NEW YORK CITY, January 9, 1904.

